# PROMP study: Effects of antidepressants during pregnancy, a retrospective cohort (2006-2018)

**DOI:** 10.1192/j.eurpsy.2025.2416

**Published:** 2025-08-26

**Authors:** A. Kurtz, A. M. Kamperman, M. Sielk, H. Bijma

**Affiliations:** 1 Psychiatry, Emergis regional mental health center, Goes; 2 Psychiatry; 3 Obstetrics and Gynaecology, Division of Obstetrics and Fetal Medicine, Erasmus University Medical Center, Rotterdam, Netherlands

## Abstract

**Introduction:**

Limited research has been done regarding antidepressant use during pregnancy. Current insights show that a higher daily-dose equivalent of maternal SRI during pregnancy is associated with lower birthweight. Additionally, also large-scale registry studies show that prenatal antidepressant exposure is associated with small decreases of pregnancy duration and birthweight, and a higher risk for preterm birth, decreased birthweight, neonatal hospitalization and postnatal neonatal withdrawal symptoms. In these studies, no differences were found regarding PPHN (Persistent Pulmonary Hypertension of the Newborn) and congenital abnormalities. Thus far, research has been done on a general population level. This study is aimed at a specific high risk population group, which makes this study much more relevant for clinical practice.

**Objectives:**

Gain insight into the effects of antidepressants during pregnancy and how they are prescribed. Specifically, the effects on birthweight and pregnancy duration, and whether the antidepressants are prescribed according to the daily-dose equivalent and are prescribed on-label.

**Methods:**

This is a retrospective clinical cohort consisting of women who between 2006 and 2018 gave birth in the Erasmus MC University Hospital, because of a medical or psychiatric referral. All women were treated by the multidisciplinary pregnancy and psychiatry outpatient clinic in the Erasmus MC, were diagnosed with a DMS diagnosis, and for this specific study were selected for antidepressant use. Birth weights were categorized against the Dutch Perined birth registry. We estimated the association of pregnancy duration with the daily-dose equivalent.

**Results:**

Of the 1372 births from mothers with a DSM diagnosis, 323 unique births were selected for antidepressant use during pregnancy. No effects were found regarding birthweight and pregnancy duration. There was a non-significant effect on birthweight: Kruskall-Wallis test statistic (3): 0,962, with a p-value of 0,810. And a non-significant effect on pregnancy duration using linear regression: standardized beta: -0,028, with a p-value of 0,654. The antidepressants, which included SSRIs, SNRIs, and TCAs, were described off-label in 26% of the cases. As a whole, the antidepressants were prescribed lower than the daily-dose equivalent, with a total average of 0.69.

**Conclusions:**

The fact that no effects were found on the use of antidepressants in women with a DSM-diagnosis on birthweight and pregnancy duration, is a reassuring finding. Certainly when taking into account the previous studies, that showed lower birthweights and higher risk for pre-term birth when using antidepressants during pregnancy. This might affect how antidepressants will be prescribed for pregnant women in the future. It is however remarkable, that antidepressants aren’t always prescribed on-label, and not always according to the recommended daily-dose.

**Disclosure of Interest:**

None Declared

